# An increase in non-toxigenic *Corynebacterium diphtheriae* cases in Philadelphia, PA, USA

**DOI:** 10.1128/asmcr.00051-26

**Published:** 2026-09-03

**Authors:** Elizabeth Novick, Eleanor Cheung, Macy Matheson, Courtney E. Comar

**Affiliations:** 1 Division of Infectious Diseases and Environmental Medicine, Department of Internal Medicine, Thomas Jefferson University 6559, Philadelphia, Pennsylvania, USA; 2 Sidney Kimmel Medical College, Thomas Jefferson University 6559, Philadelphia, Pennsylvania, USA; 3 Department of Pathology and Genomic Medicine, Thomas Jefferson University 6559, Philadelphia, Pennsylvania, USA; Pattern Bioscience, Austin, Texas, USA

**Keywords:** unstable housing, substance use disorder, urban epidemiology, *Corynebacterium diphtheriae*

## Abstract

Non-toxigenic *Corynebacterium diphtheriae* is increasingly recognized as a clinically important pathogen, particularly in association with chronic, non-healing cutaneous ulcers and, in some cases, invasive disease such as septic arthritis, osteomyelitis, bacteremia, and endocarditis. Prior reports suggest that individuals with substance use disorder and unstable housing are at heightened risk, but data characterizing the spectrum of disease in the United States remain limited. At our inner-city hospital system in Philadelphia, which serves a population with high rates of substance use and homelessness, we observed an increase in non-toxigenic *C. diphtheriae* isolates beginning in 2021. We conducted a retrospective chart review of all patients (35 unique patients, 38 discreet admissions) with cultures yielding non-toxigenic *C. diphtheriae* from 2021 through 2024, collecting demographic, clinical, and microbiological data. Most isolates out of the 38 discreet admissions (68.4%) were from skin and soft tissue sources, while 23.7% were from blood cultures. The majority (86.8%) of isolates from the 38 admissions were biovar mitis, with biovar gravis identified in 13.2%. Most patients had a history of substance use disorder (81.6%) and unstable housing (60.5%). These findings suggest that non-toxigenic *C. diphtheriae* may not be a benign colonizer in this population and should be considered a potential pathogen, particularly in patients with wounds and social risk factors. Further research is needed to clarify its clinical significance and to guide treatment decisions, including antimicrobial selection.

## INTRODUCTION

Toxin-producing *Corynebacterium diphtheriae*, a gram-positive bacillus (GPB), is the etiologic agent of diphtheria and remains an important cause of morbidity worldwide. In recent years, however, non-toxigenic *C. diphtheriae* has increasingly been recognized as a clinically significant pathogen, particularly among vulnerable populations such as individuals experiencing unstable housing or substance use disorders (1–7). Despite this emerging recognition, data describing the clinical spectrum of *C. diphtheriae* infections in the United States remain limited. In the available literature, non-toxigenic strains are most commonly associated with chronic, non-healing cutaneous ulcers but have also been implicated in invasive infections, including septic arthritis, osteomyelitis, bacteremia, and endocarditis (4–14).

Our hospital system, located in Philadelphia, Pennsylvania, an epicenter of the urban opioid epidemic in the United States, serves a population with high rates of injection drug use and homelessness. Beginning in 2021, we anecdotally observed an increase in the isolation of *C. diphtheriae* from clinical specimens in our microbiology laboratory. This observation prompted a systematic retrospective investigation of *C. diphtheriae* isolates identified at our institution between 2021 and 2024, which we present here.

## MATERIALS AND METHODS

This study was reviewed by the Thomas Jefferson University Institutional Review Board and was granted exempt status (iRISID-2024-1697). A list of positive *C. diphtheriae* cultures from 2018 to 2024 was generated using the “My Reports” function in the Epic electronic health record. All standard bacterial culture types were queried for *C. diphtheriae*. This report was used to identify eligible isolates and associated patients. All cultures reviewed were from two hospitals in Philadelphia, PA, totaling 1,094 beds, and were from the emergency department or inpatient encounters. Species-level identification was performed using matrix-assisted laser desorption/ionization time-of-flight mass spectrometry (MALDI-TOF MS) (Bruker MS). Identification of coryneform gram-positive bacilli (GPB) is performed for all blood cultures, sterile specimen sources, and specimens collected in the operating room (OR). Whether identification of a coryneform GPB occurs on other culture types where they may be considered normal flora (non-sterile and non-OR collected specimens, respiratory specimens) depends on the quantity of the organism (if heavy growth, pure growth, or predominating growth), how mixed the culture is with other normal flora, and the extent of experience of the bench microbiologist reading the culture and whether they can recognize coryneform GPB morphology (newly trained bench microbiologists may use MALDI-TOF more frequently to determine if an organism is part of the normal flora). During the study period, our protocols did not significantly change. Susceptibility testing was only performed if requested by the clinical team. As part of routine public health surveillance, one isolate per patient encounter was submitted to the Centers for Disease Control and Prevention (CDC) for toxin testing by Elek test. CDC reports included toxin results and biovar determination (Biomérieux API). An electronic health record review was conducted for all patients with positive cultures, and demographic, clinical, and microbiologic variables were abstracted.

## RESULTS

We observed an increase in *C. diphtheriae* isolation in our clinical microbiology laboratory from 2021 to 2024, with 43 culture isolations during this time compared to zero isolations from 2018 to 2020 (Table 1). All isolates submitted to the CDC were confirmed to be non-toxin-producing (non-toxigenic). The majority (33/38 admissions, 86.8%) of *C. diphtheriae* isolates were biovar mitis, with biovar gravis identified in 13.2% of the admissions. Clinical and demographic data were reviewed for 38 discreet hospital admissions corresponding to 35 unique patients (Table 2). The median age of patients at admission was 43.5 years, and a majority (76.3%) were male. History of substance use disorder was high (81.6% of admissions), and 63.2% of patients reported active injection drug use at the time of admission. A majority (60.5%) of the patients were unhoused at the time of admission. History of hepatitis C infection and chronic wounds at the time of admission were also high (63.2% and 71.1%, respectively). Skin and soft tissue cultures accounted for most isolate sources. White blood cells were observed in at least one Gram stain from each admission from skin and soft tissue cultures, but GPB were only observed 53.8% of the time on the Gram stain. Many cultures were polymicrobial, with common co-occurring organisms including *Staphylococcus aureus* (23/43 cultures), *Streptococcus pyogenes* (18/43 cultures), *Streptococcus dysgalactiae* (14/43 cultures), and *Arcanobacterium haemolyticum* (13/43 cultures). Culture data for each patient is summarized in Table S1. Only four *C. diphtheriae* isolates underwent antimicrobial susceptibility testing, as testing was performed only upon request of the clinical team, and all tested were susceptible to vancomycin, daptomycin, linezolid, and ciprofloxacin.

**TABLE 1:** Non-toxigenic *Corynebacterium diphtheriae* cases, admissions, and isolations in culture by year

Years	Unique patients	Discreet admissions	Total isolations in culture^*a*^
2018–2020	0	0	0
2021	2	2	2
2022	4	4	6
2023	14	15	17
2024	15	17	18
Total	35	38	43

^*a*^ Two sets of blood cultures (four bottles total) are counted as one culture.

**TABLE 2:** Demographic and clinical characteristics of patients with non-toxigenic *Corynebacterium diphtheriae* infection with discreet admissions (*N* = 38), Philadelphia, PA, 2021–2024

Characteristic	No. (%)
Median age, years (range)	43.5 (24–66)
Sex	
Female	9 (23.7)
Male	29 (76.3)
Medical history	
HIV	4 (10.5)
History of hepatitis C infection	24 (63.2)
Diabetes mellitus	2 (5.3)
Chronic wounds	27 (71.1)
Housing status	
Currently unhoused	23 (60.5)
Stable housing	15 (39.5)
Drug use	
History of substance use disorder	31 (81.6)
Active IV drug use	24 (63.2)
Active non-IV drug use	3 (7.9)
Lifetime IV drug use	31 (81.6)
Specimen source	
Skin or soft tissue	26
*C. diphtheriae* only	0 (0.0)
Polymicrobial	26 (100.0)
White blood cells seen on Gram stain	26 (100.0)
GPB seen on Gram stain	14 (53.8)
Sputum	3
*C. diphtheriae* only	0 (0.0)
Polymicrobial	3 (100.0)
Blood	9
*C. diphtheriae* only	3 (33.3)
Polymicrobial	6 (66.7)
Multiple bottles (out of 4) positive	4 (44.4)
Outcomes	
30-day readmission	14 (36.8)

## DISCUSSION

This retrospective case series of non-toxigenic *Corynebacterium diphtheriae* infections, encompassing 38 discreet admissions for 35 unique patients over the course of a 3-year period at our inner-city Philadelphia hospital, is consistent with prior reports showing increasing rates of non-toxin-producing *C. diphtheriae* infections primarily affecting vulnerable patient populations, including those with unstable housing and substance use disorder (1–3, 15). Due to stability of laboratory identification protocols, the increase in *C. diphtheriae* isolates likely reflects a true epidemiologic increase rather than improved laboratory identification (2). The recovery of two different biovars (mitis and gravis) suggests that this may be a multiclonal outbreak, although whole-genome sequencing would provide further clarity on the clonality of the isolates and if there were any potential transmission chains in our patients.

This study has several limitations, primarily due to the retrospective nature of the study. All data were collected retrospectively from the electronic health record, which may impact completeness and accuracy. For example, information including travel history, Tdap vaccination status, and whether antibiotic treatments were broad or targeted to include coverage of *C. diphtheriae* was not readily available for all patients to include in the study. As a retrospective case series review, we did not have a matched control group to compare outcomes. Additionally, because of the retrospective review, we could not perform whole-genome sequencing or expanded susceptibility testing, limiting insights into transmission dynamics and resistance patterns. Notably, other *Corynebacterium* species which can harbor diphtheria toxin, *Corynebacterium ulcerans* and *Corynebacterium pseudotuberculosis*, were not included in this study. Unlike *C. diphtheriae*, our laboratory protocols do not include automatically reporting the species-level identification of these two species regardless of source. Furthermore, we do not automatically send these two species for toxin testing. Future investigations should determine whether *C. ulcerans* and *C. pseudotuberculosis* have similar epidemiological patterns as non-toxigenic *C. diphtheriae*. Consistent with previous studies, many of the *C. diphtheriae* isolates were recovered from polymicrobial cultures (2, 15, 16). The frequent co-isolation of *Staphylococcus aureus*, beta-hemolytic *Streptococci*, and *Arcanobacterium haemolyticum* complicates the determination of the pathogenic role of non-toxigenic *C. diphtheriae*, particularly in chronic wounds (3, 17). Rising rates of chronic, non-healing wounds among people using drugs, particularly related to xylazine contamination in the drug supply, may contribute to the organism’s emergence in this population (18, 19).

Further research is needed to understand the pathogenic potential of non-toxigenic *C. diphtheriae*, particularly to distinguish colonization from infection in non-sterile sites and to characterize the risk for invasive disease. Notably, because non-toxigenic *C. diphtheriae* is often isolated with other pathogens, this highlights the complexity of understanding its role in pathogenesis. Furthermore, laboratory practices for identification of coryneform GPB widely vary. Indeed, a limitation of our study is that some infections with mixed cultures from non-sterile sources (respiratory and non-OR collected wound cultures) may have been missed if identification by MALDI-TOF of a coryneform GPB was not performed. Our study highlights that identification to the species level of coryneform GPBs, even in polymicrobial cultures or single positive blood culture bottles, is important to identify *C. diphtheriae*. In our laboratory, if *C. diphtheriae* is identified, it is not considered a blood culture contaminant (even if identified in only one blood culture bottle within a set). As MALDI-TOF has become widely available for bacterial identification, laboratories may need to consider their policies on identification of coryneform GPBs to the species level, especially if their laboratory serves a large patient population with epidemiologic risk factors for non-toxigenic *C. diphtheriae*, such as substance use or unstable housing. Additional studies should also evaluate antimicrobial susceptibility patterns, especially for oral agents suitable for outpatient treatment, as large studies of *Corynebacteria* susceptibility testing from North America often exclude *C. diphtheriae* (20–22), although a recent study from McTaggart et al. (15) included susceptibility data from 49 isolates and demonstrated little antibiotic resistance apart from intermediate penicillin susceptibility in most isolates.

In summary, this case series adds to the growing evidence that non-toxigenic *C. diphtheriae* is increasingly encountered in vulnerable patients, including those with unstable housing and with substance use disorders. Further work is needed to elucidate the reasons for its emergence, define its pathogenic role, and guide clinical management.

## Data Availability

The data underlying this article will be shared on reasonable request to the corresponding author.

## SUPPLEMENTAL MATERIAL

Table S1 asmcr.00051-26-s0001.xlsx Description of culture data.

ASM does not own the copyrights to Supplemental Material that may be linked to, or accessed through, an article. The authors have granted ASM a non-exclusive, world-wide license to publish the Supplemental Material files. Please contact the corresponding author directly for reuse.
